# A Two-Step Process of Nitrous Oxide before Carbon Dioxide for Humanely Euthanizing Piglets: On-Farm Trials

**DOI:** 10.3390/ani8040052

**Published:** 2018-04-04

**Authors:** Rebecca K. Smith, Jean-Loup Rault, Richard S. Gates, Donald C. Lay

**Affiliations:** 1 Department of Animal Sciences, Purdue University, West Lafayette, IN 47907, USA; 2 Institute of Animal Husbandry and Animal Welfare, University of Veterinary Medicine, A-1210 Vienna, Austria; Jean-Loup.Rault@vetmeduni.ac.at; 3 Department of Agriculture and Biological Engineering, University of Illinois, Urbana, IL 61801, USA; rsgates@illinois.edu; 4 USDA-ARS, Livestock Behavior Research Unit, West Lafayette, IN 47907, USA; Don.Lay@ARS.USDA.GOV

**Keywords:** on-farm killing, euthanasia, neonatal piglet, carbon dioxide, nitrous oxide

## Abstract

**Simple Summary:**

The current approved method of using carbon dioxide (CO_2_) to euthanize newborn piglets is raising animal welfare concerns on whether the method is truly humane. A new form of euthanasia that is humane, practical, and socially acceptable is needed. Nitrous oxide (N_2_O), also known as laughing gas, has been shown to induce narcosis in piglets. We used a novel two-step system of exposing compromised piglets for six minutes to N_2_O followed by carbon dioxide and compared it to using CO_2_ alone. After exposure to nitrous oxide, all piglets lost posture, a sign of the onset of loss of consciousness, before being exposed to CO_2_ when they showed behavioral distress. On-farm use of a two-step method reduced the amount of time the piglets were exposed to CO_2_ but did not reduce the amount of distressful behaviors. Therefore, the results do not support the hypothesis that using N_2_O in a two-step system is more humane than CO_2_ alone.

**Abstract:**

Current methods of euthanizing piglets are raising animal welfare concerns. Our experiment used a novel two-step euthanasia method, using nitrous oxide (N_2_O) for six minutes and then carbon dioxide (CO_2_) on compromised 0- to 7-day-old piglets. A commercial euthanasia chamber was modified to deliver two euthanasia treatments: the two-step method using N_2_O then CO_2_ (N_2_O treatment) or only CO_2_ (CO_2_ treatment). In Experiment 1, 18 piglets were individually euthanized. In Experiment 2, 18 groups of four to six piglets were euthanized. In the N_2_O treatment, piglets lost posture, indicating the onset of losing consciousness, before going into CO_2_ where they showed heavy breathing and open-mouth breathing; whereas piglets in the CO_2_ treatment did not lose posture until after exhibiting these behaviors (*p* ≤ 0.004). However, piglets in the N_2_O treatment took longer to lose posture compared to the CO_2_ treatment (*p* < 0.001). Piglets in the N_2_O treatment displayed more behavioral signs of stress and aversion: squeals/minute (*p* = 0.004), escape attempts per pig (*p* = 0.021), and righting responses per pig (*p* = 0.084) in a group setting. In these regards, it cannot be concluded that euthanizing piglets for 6 min with N_2_O and then CO_2_ is more humane than euthanizing with CO_2_ alone.

## 1. Introduction

The first days of a piglet’s life are critical for its survival. There is an average of 9.7% pre-weaning mortality in indoor pig farming systems in the United States [1]. Nearly half of these deaths are due to crushing by the sow while a large percentage of the rest is due to scours and failure to thrive [1]. The morbid piglets deserve a quick, painless death to end their suffering: euthanasia [2]. The American Veterinary Medicine Association (AVMA) guidelines for the euthanasia of neonatal piglets include carbon dioxide (CO_2_), mixtures of CO_2_ and argon (Ar) or nitrogen (N_2_), carbon monoxide (CO), inhaled anesthetics, purpose-built nonpenetrating captive bolt, anesthetic overdose, and blunt force trauma [3]. Blunt force trauma and CO_2_ are widely used on-farm, however there is public concern on the humaneness of these methods. For a method to be humane, it should minimize pain and distress, induce a rapid loss of consciousness, and achieve death quickly and consistently [3,4]. Blunt force trauma is not visually appealing and if unsuccessful the first time, the piglet can suffer. Carbon dioxide chambers are widely used; however, CO_2_ is highly aversive to swine at 20% [5] and 30% [6] or greater. At 70 and 90% CO_2_, Velarde et al. observed piglets refusing to enter a crate with CO_2_ voluntarily, attempting to retreat, attempting to escape, and an increased time to enter the crate [7]. Sadler et al. euthanized piglets with 100% CO_2_ or a mixture of 50:50 CO_2_:Ar at different flow rates and observed escape attempts, open-mouth breathing, and righting responses [8]. These behaviors are indicators of aversion. When CO_2_ was mixed with N_2_ in three combinations (70% N_2_:30% CO_2_, 80% N_2_:20% CO_2_, and 85% N_2_:15% CO_2_), the majority of pigs attempted to retreat, attempted to escape, and vocalized in all three treatments concluding that pigs show aversion to CO_2_ at 15–30% in N_2_ [9]. Therefore, there is a need to develop a better method of euthanizing neonatal piglets.

The current gas euthanasia methods are advantageous because multiple animals can be euthanized at once and it offers an accommodating approach, beneficial from a psychological point of view for the caretakers, and for biosecurity as there is less need for handling the animal. To exploit these advantages, a new gas method that uses a non-aversive gas may prove to be a better approach. Nitrous oxide (N_2_O), commonly known as laughing gas, is widely used in human medicine as an anesthetic. When humans are exposed to increasing concentrations of N_2_O in oxygen, they show a decrease in response to a painful stimulus [10]. N_2_O has also been shown to induce narcosis in piglets [11]. Rault et al. [5] explored the possibility of using N_2_O as a euthanasia method for neonatal piglets. Using a free-choice, approach-avoidance test, they assessed the aversiveness of different gas mixtures and then euthanized piglets with the mixtures to determine how effective and humane they were. All gas mixtures that contained CO_2_ were aversive to piglets, however the mixture with N_2_O was least aversive. When euthanizing, a mixture of 60% N_2_O and 30% O_2_ caused the piglets to fall recumbent and become unresponsive, at which point the piglet was moved to a different chamber with CO_2_. This process took about 12 min longer than the gas mixtures with CO_2_. They concluded that a two-step procedure of exposing piglets to a mixture of N_2_O and O_2_ followed by exposure to CO_2_ may be a more humane way to euthanize piglets than CO_2_ alone [5].

Rault et al. experimented with 10- to 14-day-old piglets and their perception of different N_2_O concentrations in a place-avoidance test and used electroencephalography (EEG) to validate the effectiveness and humaneness of nitrous oxide to induce loss of consciousness. The results showed that N_2_O is much less aversive than CO_2_ and 90% N_2_O can euthanize piglets [12]. From the knowledge of these previous experiments, an experiment was designed that combined N_2_O and CO_2_ in a two-step process to see if it is a more humane method than CO_2_ alone and used younger piglets that were ill or compromised in a farm setting to get a more realistic approach. This experiment was comprised of two parts. The goal of Experiment 1 was to evaluate an individual piglet’s welfare when being euthanized. Experiment 2 assessed piglet welfare in groups on a commercial farm. The goal of Experiment 2 was to determine if the two-step method would work in a practical manner on a large commercial farm where they must euthanize many piglets daily. We hypothesized that N_2_O followed by CO_2_ would be less distressful, when compared to CO_2_ alone, as pigs would lose posture, an indicator that the piglet has started to enter a state of unconsciousness, before being exposed to CO_2_. We predicted that a two-step euthanizing procedure with N_2_O followed by CO_2_ would decrease distressful behaviors (heavy breathing, open-mouth breathing, squeals, escape attempts, neck stretches, and righting responses).

## 2. Materials and Methods

### 2.1. Animals and Housing

#### 2.1.1. Experiment 1

All research was approved by the Purdue University Animal Care and Use Committee (PACUC, 1410001143). The piglets were progeny of a commercial crossbred line housed at the Purdue University Animal Science Research and Education Center. Sows and their piglets were housed in conventional farrowing crates. Only compromised piglets 0–7 days of age that were fated to be euthanized by the Purdue University farm staff or the experimenters were used. Compromised piglets included severely injured or non-ambulatory piglets with the inability to recover, piglets that were not gaining weight and had a body condition score of one, and sick piglets that did not show adequate improvement after two days of intensive care [4]. The justification of using this age is that this type of piglet represents the population of interest, as this is when morbidity and the need for euthanasia is greater in production settings. Piglets were not screened for their halothane genotype, which may influence their sensibility to gas changes [7], although the proportion of halothane gene in this herd is expected to be very low. An attempt was made to use only one piglet per sow to avoid bias; however, when two piglets were euthanized from the same sow they were subject to different treatments. An attempt was also made to have an equal representation of sexes in each treatment and from the 18 piglets used; nine were female and nine were male.

**Figure 1:**
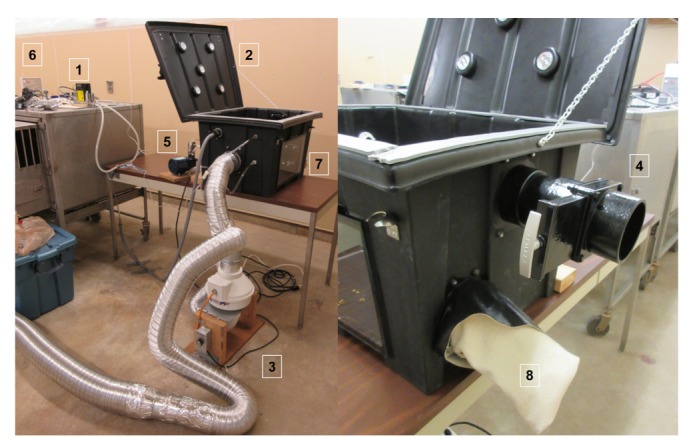
Setup of the gas chamber for Experiment 1. The two treatment gases (CO_2_ and N_2_O) went from the tanks to the mass flow controllers (1), into the chamber box (2), and then out of the chamber with help from a ventilator (3) and a gate valve that allowed the system to be flushed (4). A vacuum pressure pump (5) kept the system under a slight negative pressure that was measured by a manometer (6). Acrylic glass (7) allowed visualization of the piglets. To check for palpebral reflexes there was a glove assembly (8) for reaching into the chamber.

A euthanasia gas chamber (Figure 1, 61 cm × 38 cm × 46 cm, Euthanex^®^ AgPro^TM^, NutriQuest Inc., Mason City, IA, USA) was modified so that N_2_O and CO_2_ could be delivered to the chamber in a two-step procedure at regulated levels. A ribbed rubber mat (MT4000019, Multy Home^TM^, Toronto, ON, Canada) was placed in the bottom of the box to prevent slipping and allow for easy clean up. The chamber top was fitted with weather stripping (38351, M-D Building Products, Oklahoma City, OK, USA) to achieve a tight seal. Battery operated lights were attached to the lid to provide adequate lighting for video recording. One sidewall was modified with entry and exit holes to accommodate gas filling and extraction. Gas from the CO_2_ and N_2_O tanks went through their respective regulators, clear vinyl tubing (0.95 cm ID × 1.27 cm OD, Sioux Chief, Kansas City, MO, USA), a plastic on/off switch valve, mass flow controller (GFC47, Aalborg Instruments & Controls, Inc., Orangeburg, NY, USA), more tubing (POLYAIR^®^ 1.27 cm ID, K1138, Kuri Tec, Brantford, ON, Canada), and into the chamber. Both treatments followed the standard recommendation of gas concentration per time [3], namely a 25% replacement rate per minute accomplished using the mass flow controllers. Gas extraction consisted of flowing out of the box into more clear vinyl tubing attached to a power ventilator (FR100, Fantech, Lenexa, KS, USA) and a 10.16 cm spiral metal duct pipe (81010416, Heating and Cooling Products, Mt. Vernon, OH, USA) that led to the outside of the building. Across from this tube in the box, there was a 7.62 cm hole with a PVC gate valve attached that opened and closed to the outside. At the end of the experiment the gate valve could be opened to flush the box of all the gases into the ventilator. This ensured proper evacuation of excess N_2_O from the chamber area and allowed workers safe access during testing. Another tube was attached to a vacuum/pressure pump (Air Cadet 7530-40, Cole-Parmer, Vernon Hills, IL, USA) that maintained the chamber under slight negative pressure. A manometer (Mark ll #25, Dwyer Instruments Inc., Michigan City, IN, USA) was used to make sure the chamber was under negative pressure during the experiment. The front side of the box contained acrylic glass for observations (Figure 1) and the right side of box contained a shoulder length glove assembly (Ansell Neox^®^, 1559, Northern Safety Co., Inc., Utica, NY, USA) to allow the experimenter to check the piglet’s reflexes. A camera (KPC-N502NUB, KT&C, Fairfield, NJ, USA) was positioned in front of the acrylic glass and video was recorded using video management software (GeoVision Network Video Recorder GV-NVR, Taipei, Taiwan).

Before euthanizing any piglets, gas was turned on to make sure no leaks were heard and ran for 20 min with a temperature data logger (HOBO^®^ U12-112, Onset, Bourne, MA, USA) in the chamber. The temperature averaged 20.77 ± 3.06 °C (mean ± standard error). The gas was then flushed from the chamber before testing started.

#### 2.1.2. Experiment 2

The piglets were progeny of a commercial crossbred line housed at a local producer’s farm. Piglets 0–6 days of age designated to be euthanized by the farm staff were used. Since piglets were being euthanized in groups and experimenters were blind to the piglet’s identification, the attempt to not have siblings in the same treatment could not be made. In the 18 groups, there was a total of 91 piglets euthanized: 47 females and 44 males.

**Figure 2:**
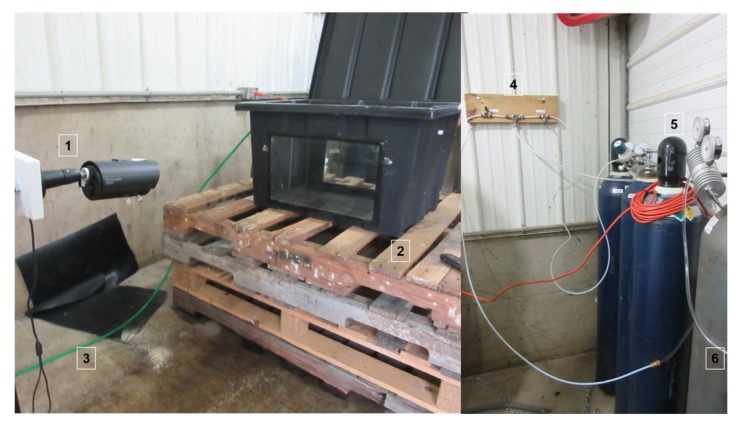
Setup of the gas chamber for Experiment 2. Setup was similar to Experiment 1 except there were two cameras (1) and two acrylic glass observation windows (2). Instead of a ventilator, the vacuum pump created negative pressure and the air it took out was routed through a tube (3) to the outside. The on/off valves (4), tubing, and tanks (5) were similar. However, there was only one tube (6) that went to one mass flow controller and then into the box, instead of two mass flow controllers as was used in experiment 1.

The gas chamber and setup were similar to Experiment 1 with a few exceptions. The box had no glove assembly, no gate valve, no lights on the lid, no manometer, and no ventilator. Instead, the vacuum acted as the ventilator and kept a slight negative pressure. The air the vacuum pumped out was routed out of the building with tubes for handler safety. There were also two acrylic glass windows (along the sides of longest length of the box) and two cameras (Figure 2).

### 2.2. Overall Experimental Design

Piglets were subject to one of two euthanasia treatments: gradual fill of CO_2_ or gradual fill of N_2_O for six min (360 s) followed by CO_2_ until clinical death. The amount of time that N_2_O was to be used before CO_2_ was determined from EEG results previously done by Rault et al., with the six min time frame being defined as the time when the last of all the piglets tested with N_2_O exhibited a transitional EEG pattern [13]. To assure most piglets reached this state of transitional EEG before CO_2_ was applied, the longest duration of N_2_O exposure was used. N_2_O and CO_2_ were delivered at a 25% replacement rate per minute. Nitrous oxide gas concentrations were validated using indirect measurements of O_2_ and CO_2_ because N_2_O concentrations at the levels used could not be directly measured. Specifically, lab commissioning of the system was used with O_2_ sensors to record O_2_ displacement at these rates, with N_2_ as the test gas. Oxygen concentration was below 5% within 4.5 min in four replicates with very consistent and repeatable responses. A system analysis of the N_2_ fill response indicated a time-to-95% of steady-state of 490–517 s (mean 498.5 s), which is in reasonable agreement with a 4 min time constant predicted for a well-mixed chamber.

### 2.3. Procedures

#### 2.3.1. Experiment 1

When a piglet was found that needed to be euthanized, it was collected and the rectal temperature, weight, sex, age, pig identification, and reason for euthanizing were recorded. Treatment had been randomly assigned for 18 piglets before any piglets were euthanized using a random number generator (www.random.org). Videos were captured and then analyzed with a commercial software program (The Observer XT 11, Noldus, Wageningen, The Netherlands). Two categories of behavior were observed (Table 1): posture (stand, lateral lying, ventral lying, sit, kneel) and activity (locomotion, inactive, rooting, escape attempt, foot slipping, neck stretch, heavy breathing, open-mouth breathing, ataxic, righting response, loss of posture, paddle). The durations of the postures and latency to the activities were recorded. Vocalizations were recorded with a digital sound recorder (ICD-PX333, Sony Electronics Inc., Minato, Tokyo, Japan) that was placed inside the chamber during euthanasia. Grunts, squeals and intermediate noises (Table 1) were listened to on sound organizer software (Version 1.4, Sony Electronics Inc., Minato, Tokyo, Japan) and a count was made by ear. To determine when the pigs became unconscious, their palpebral reflex and response to pin prick on the nose was determined every 30 s after the piglet assumed a loss of posture [14]. The piglet was determined dead after there were no more palpebral reflexes and breathing and gaping had ceased. The CO_2_ was turned off at this time. The total time with CO_2_ and time to no reflex response was recorded with a standard stop watch.

#### 2.3.2. Experiment 2

Piglets were euthanized in groups of four to six with a procedure similar to Experiment 1. The piglets had enough room in the box to stand on all four legs, not be on top of each other, and the ability to move around. With more piglets in the box, it was not practical to get every single behavior and palpebral reflex as in Experiment 1, so only some of the activity behaviors were collected and processed (Table 1). Vocalizations were again captured but since there were multiple piglets all making noise at once, only squeals were quantified for the group as a whole (Table 1). The weight, sex, age, and reason for euthanizing were recorded for each piglet. The length of time CO_2_ was used was recorded using a stop watch. The treatment for each group was predetermined and randomized by a random number generator (random.org).

### 2.4. Data Processing

All raw data points were taken from the behavioral software. The program provides relative times and durations in seconds. In Experiment 1, total time durations were added up for standing, lateral lying, ventral lying, sitting, kneeling, locomotion, inactivity, and total time for the entire procedure. The amount of time it took for a state to take place and the frequency of events were calculated and latencies are shown for ataxia, heavy breathing, open-mouth breathing, gaping, loss of posture, and first paddle bout. Two of the piglets did not have data for latency to ataxia due to ambulatory problems. One piglet’s data was omitted from analysis as it was older than seven days of age; thus, data for eight CO_2_ and nine N_2_O were analyzed. The number of squeals, intermediate vocalizations, and grunts were divided by the total duration of the procedure. One piglet did not have vocalization data for the experiment because of a technical issue; thus, data for eight CO_2_ and eight N_2_O were analyzed.

In Experiment 2, five of the piglets did not stand at all during the procedure as they were too sick or injured, so behavior was not collected on them and the total number of piglets was adjusted: 91 piglets euthanized and 86 piglets standing. Behaviors collected, as indicated in Table 1, included latencies to first piglet heavy breathing, first piglet open-mouth breathing, first piglet gaping, all piglets gaping, first piglet that lost posture, all piglets that lost posture, first paddle bout, first piglet’s last movement, and last piglet’s last movement. Total time duration for the whole procedure was recorded. The number of escape attempts and righting attempts were divided by the number of piglets in the box. The age and weight of the piglets were averaged for each group. Squeals were divided by the total time.

### 2.5. Statistical Methods

Data were analyzed in SAS (version 9.4., SAS Institute Inc., Cary, NC, USA). All data were checked for normality and homogeneity of variance prior to analysis. The mean ± standard error is shown for all data.

In Experiment 1, the experimental unit was the piglet. Normal data were analyzed as a mixed model analysis of variance with treatment and sex as fixed effects. The piglet’s body temperature, weight, and age served as covariates. Log transformations were performed as needed. Normal data included: time to no reflexes, log of grunts/minute, log of standing duration, ventral lying duration, log of latency to heavy breathing, log of latency to loss of posture, log of latency to first paddle bout, log of total time for the entire procedure, age, body temperature, and weight. All other data were non-normal and were analyzed using the Wilcoxon-Mann-Whitney test statistic.

In Experiment 2, the group of piglets was the experimental unit. Normal data were analyzed as a mixed model analysis of variance with treatment as a fixed effect. Log transformation was performed as needed. Normal data included: CO_2_ total time, number of males, number of females, group average day of age, log of latency to first piglet losing posture, latency to first piglet’s paddle bout, latency to first piglet’s last movement, and total time for the entire procedure. All other data were non-normal and were analyzed using the Wilcoxon-Mann-Whitney test statistic. Data were considered different for *p* < 0.05 and exhibited a trend when 0.05 < *p* < 0.10.

An observation was made in the N_2_O treatments, for both Experiments 1 and 2, that heavy breathing and open-mouth breathing, recorded as latencies, happened only when CO_2_ was present. So, for the latencies in the N_2_O treatment, six min (360 s) were subtracted to allow comparisons to be made between the latencies post-N_2_O and the CO_2_ treatment, thus only when pigs are exposed to CO_2_. This comparison allowed for isolation of the effect of CO_2_ only, and could determine if pre-exposure to CO_2_ impact latencies for these behaviors. For both experiments, the entire latency data were analyzed first, then six min (360 s) were subtracted from the N_2_O treatment data and analyzed again to compare the N_2_O treatment (post-N_2_O) against the CO_2_ treatment. The post-N_2_O data vs. CO_2_ treatment data were analyzed as according to above. In Experiment 1, normal data included latency to open-mouth breathing post-N_2_O and latency to gaping post-N_2_O. In Experiment 2, normal data included latency to heavy breathing post-N_2_O, latency to open-mouth breathing post-N_2_O, latency to first piglet gaping post-N_2_O, latency to all piglets gaping post-N_2_O, latency to first piglet’s last movement post-N_2_O, and latency to last piglet’s last movement post-N_2_O. All other post-N_2_O data were non-normal and analyzed using the Wilcoxon-Mann-Whitney test statistic.

## 3. Results

### 3.1. Experiment 1

The reasons for euthanizing the piglets were not balanced across the two treatments. In the CO_2_ treatment, six were malnourished (emaciated, ribs apparent), one was injured, and one was small. In the N_2_O treatment, one piglet was malnourished, five were injured, one was small, and two were sick. The sex of the piglets was balanced across the two treatments: N_2_O had four males and five females while CO_2_ had four males and four females. Piglets in the N_2_O treatment averaged one day of age older than the CO_2_ treatment (*p* = 0.034; Table 2). Body temperature and weight of the piglets averaged the same for each treatment (*p* > 0.05; Table 2). The total amount of time CO_2_ gas was run and the total minutes it took for the entire procedure also averaged the same for each treatment (*p* > 0.05; Table 2). Latency to no palpebral reflex took an average of 4 min longer for N_2_O piglets compared to CO_2_ piglets (*p* < 0.001; Table 2).

#### 3.1.1. Durations and Latencies

**Figure 3:**
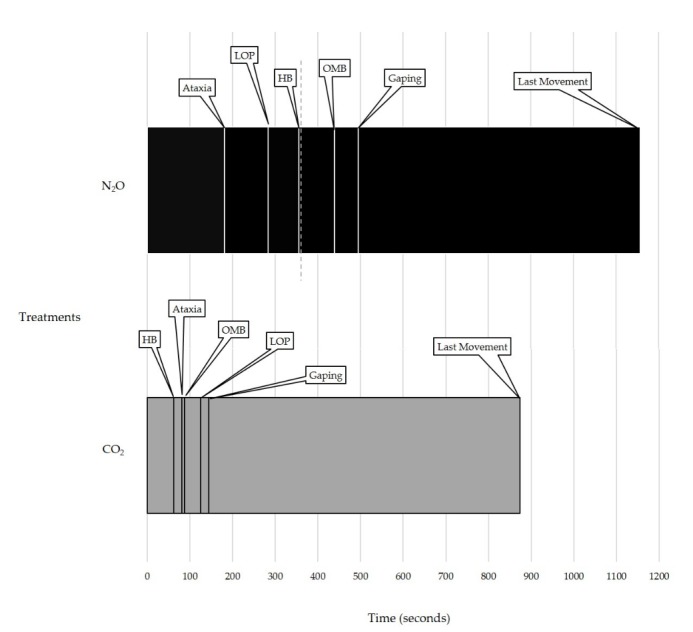
Sequence of latency behaviors for individual piglets in Experiment 1 for each treatment: N_2_O for six min followed by CO_2_ (N_2_O treatment) or just CO_2_ (CO_2_ treatment). Using the means of each latency, a timeline was created to highlight the difference in the order in which the behaviors occurred. The N_2_O treatment has an additional dotted line at six min (360 s) to represent when the N_2_O was shut off and CO_2_ was turned on. HB = heavy breathing, OMB = open-mouth breathing, LOP = loss of posture.

Piglets spent the same amount of time standing, lying, sitting, kneeling, locomoting, or being inactive in both treatments (*p* > 0.05; Table 3). In the N_2_O treatment, piglets took an average of four min longer before reaching ataxia (*p* = 0.021; Table 3) and twice as long to loss of posture (*p* = 0.004; Table 3). Ataxia and loss of posture took place before piglets were switched from N_2_O to CO_2_. Piglets also took an average of five to six min longer before beginning to show heavy breathing, open-mouth breathing, and gaping (*p* < 0.05; Table 3), during the N_2_O treatment compared to the CO_2_ treatment. Piglets in the N_2_O treatment, displayed these behaviors when CO_2_ was present. Latency to the first paddle bout took the same amount of time for both treatments (*p* = 0.370; Table 3), however only seven of the 17 piglets exhibited at least one paddle bout: three in the CO_2_ treatment and four in the N_2_O treatment. The latency behaviors did not occur in the same order for both treatments (Figure 3).

#### 3.1.2. Behavioral Events

Piglets in both treatments exhibited the same number of distressful behaviors; escape attempts, neck stretches, and righting responses (*p* > 0.05; Table 4). The number of paddle bouts and foot slips displayed in each treatment were also similar (*p* > 0.05; Table 4). In the N_2_O treatment, piglets showed a trend to have more rooting bouts (*p* = 0.095; Table 4). Squeals, intermediate vocalizations, and grunts were observed at the same rate in both treatments (*p* > 0.05; Table 4). Two of the piglets made no vocalizations at all (1 CO_2_, 1 N_2_O), only six of the 17 squealed (3 CO_2_, 3 N_2_O), a little over half had righting attempts (4 CO_2_, 6 N_2_O), and a little under half had escape attempts (4 CO_2_, 2 N_2_O).

### 3.2. Experiment 2

The reasons for euthanizing the piglets in this experiment were also not balanced across the two treatments. In the CO_2_ treatment, 10 piglets were malnourished, seven were injured, and 29 were small. In the N_2_O treatment, six piglets were malnourished, three were injured, and 36 were small. Genders were balanced across treatments: CO_2_ had 22 males and 24 females whereas N_2_O had 22 males and 23 females. Average day of age and average weight were similar for both treatments (*p* > 0.05; Table 5). 

Piglets in the N_2_O treatment had ten times as many squeals per minute than piglets in the CO_2_ treatment (*p* = 0.004; Table 5). Three groups did not squeal at all and eight groups had no escape attempts. Piglets in the N_2_O treatment also exhibited more escape attempts (*p* = 0.021; Table 5) and tended to have a higher number of righting responses (*p* = 0.084; Table 5) than piglets in the CO_2_ treatments. The total amount of time CO_2_ gas ran during the experiment averaged two min longer in the CO_2_ treatment compared to the N_2_O treatment (*p* < 0.001; Table 5). The total amount of time for the entire procedure averaged about three min longer for the N_2_O treatment compared to the CO_2_ treatment (*p* = 0.001; Table 5).

#### Latency Data

**Figure 4:**
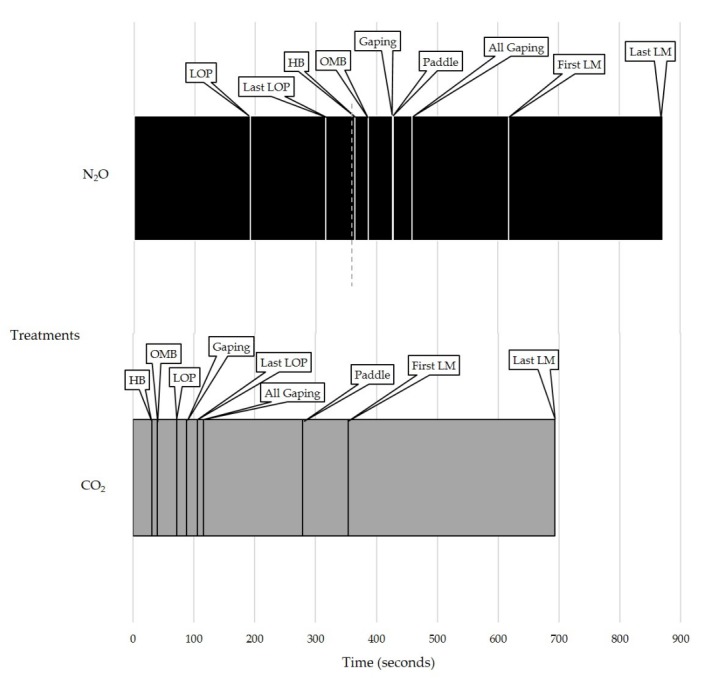
Sequence of latency behaviors for groups of piglets in Experiment 2 for each treatment: N_2_O for six min followed by CO_2_ (N_2_O treatment) or just CO_2_ (CO_2_ treatment). Using the means of each latency, a timeline was created to highlight the difference in the order in which the behaviors occurred. The N_2_O treatment has an additional dotted line at six min (360 s) to represent when the N_2_O was shut off and CO_2_ was turned on. HB = heavy breathing, OMB = open-mouth breathing, LOP = loss of posture, LM = last movement, First = first piglet, Last = last piglet.

Groups of piglets took longer to show signs of all the latency behaviors in the N_2_O treatment than the CO_2_ treatment (*p* < 0.05; Table 6). The order in which the latencies occurred was different in each treatment (Figure 4).

### 3.3. Exposure to CO_2_ Post-N_2_Os

In Experiment 1, when six min were subtracted from the latencies in the N_2_O treatment, to only look at the latencies when CO_2_ gas was running (post-N_2_O), the heavy breathing, open-mouth breathing, gaping, and paddling all happened at the same time when comparing post-N_2_O exposure to CO_2_ to CO_2_ alone (*p* > 0.05; Table 7). Ataxia and loss of posture took place when N_2_O gas was present before CO_2_ in the N_2_O treatment, so they happened four and three min quicker in the post-N_2_O treatment than in the CO_2_ treatment respectively (*p* < 0.05; Table 3).

In Experiment 2, when comparing the N_2_O treatment post-N_2_O against the CO_2_ treatment, all the latency data were now shorter in the post-N_2_O treatment (*p* < 0.05; Table 8).

## 4. Discussion

Piglets all lost posture within six min (360 s) while exposed to N_2_O, before being exposed to CO_2_. The pigs were still equally responsive to the CO_2_ gas after the gases were switched, based on similar latencies to distressful behaviors (heavy breathing and open-mouth breathing). Therefore, these results do not support that pre-exposure to gradual fill N_2_O for 6 min followed by gradual fill CO_2_, as a two-step procedure, is more humane than just gradual fill CO_2_ alone.

### 4.1. Experiment 1

Euthanizing piglets individually with CO_2_ or a combination of N_2_O for six min and then CO_2_ did not result in many significant differences. The hypothesis that exposure to N_2_O prior to exposure to CO_2_ is a more humane way to euthanize neonate piglets was only partially supported. The behavioral signs of stress and distress that were collected, squeals, escape attempts, neck stretches, and righting response, were not different between the two treatments. Since it took longer for the piglets to lose posture in the N_2_O compared to the CO_2_, they had more opportunities to display escape attempts, neck stretches, and squeals. Righting responses occurred in both N_2_O and CO_2_. Due to CO_2_ having an almost immediate effect, which causes pigs to breath heavily, it severely decreases their ability to vocalize, thus creating an unfair comparison to the behavior of the N_2_O pigs. The grunting behavior also did not differ.

There was a difference in the distressful behaviors of heavy breathing and open-mouth breathing. Heavy breathing and open-mouth breathing are signs of breathlessness which is associated with unpleasantness and compromised welfare [6,15]. Open-mouth breathing is a behavior that is typically observed before loss of posture when using CO_2_ [8,16]. The latency to heavy breathing and open-mouth breathing were longer in the N_2_O treatment than in the CO_2_ treatment, and occurred after loss of posture and after the N_2_O had been switched to CO_2_ in the chamber. This response is indicative that it is the CO_2_ specifically which causes these behaviors to occur, at least in this time frame. Piglets in the CO_2_ treatment experienced heavy breathing and open-mouth breathing before loss of posture. In humans, these ventilatory responses are due to the central and peripheral chemoreceptors detecting increases in CO_2_ in the blood. These chemoreceptors monitor the levels of CO_2_ and oxygen in the body to regulate breathing. They do not detect N_2_O so increases in N_2_O are not regulated and do not cause changes in breathing like CO_2_ [17]. For loss of posture, other authors have proposed that it may be an indicator of loss of consciousness, at least as an early sign [6,14]. Piglets in the N_2_O treatment lost posture before experiencing these distressful behaviors. Since loss of posture has been suggested to coincide with the onset of loss of consciousness, this suggests that N_2_O pigs may experience better welfare since they are exposed to CO_2_ only after they start to lose consciousness. However, it is apparent that pigs make righting responses after they lose posture which is indicative of some level of consciousness; it is not apparent to what extent the pigs may be experiencing pain or distress after loss of posture. When comparing the N_2_O treatment post-N_2_O against the CO_2_ treatment, the latencies occurred at about the same time once CO_2_ was introduced, except for ataxia and loss of posture which occurred when piglets were exposed to N_2_O. This comparison emphasized that ataxia and loss of posture occurred before heavy breathing and open-mouth breathing in the N_2_O treatment.

The lack of differences for the other parameters measured between the two treatments may be due to the piglets being so young. Neonate piglets succumb to CO_2_ and a mixture of CO_2_ and Ar faster than weaned pigs and show less signs of distress [8]. The signs of distress collected in this experiment were not observed for each individual. Two of the piglets made no vocalizations at all (1 CO_2_, 1 N_2_O), only six of the 17 squealed (3 CO_2_, 3 N_2_O), a little over half had righting attempts (4 CO_2_, 6 N_2_O), and a little under half had escape attempts (4 CO_2_, 2 N_2_O). Other euthanasia studies also recorded escape attempts in neonates and did not observe any [8,18]. Escape attempts were only observed in weaned pigs [8]. However, this may be due to their use of different CO_2_ concentrations, younger piglets, different gas treatments, different box construction, etc. Experimenting with older piglets may broaden the behavioral differences and increase the number of escape behaviors observed. In addition, euthanizing piglets in groups rather than individually may show more differences as pigs are susceptible to isolation distress [15,19].

### 4.2. Experiment 2

Piglets euthanized in groups with CO_2_ or N_2_O for six min and then CO_2_ resulted in more significant differences than the individual data. The distressful behaviors of squeals, escape attempts, and righting responses occurred with higher frequency in the N_2_O treatment compared to the CO_2_ treatment. Since the piglets in the N_2_O treatment spent more time upright, they had more time to squeal and make escape attempts during this time, so this comparison is not ideal. Squeals are associated with a negative affective state [20]. More squeals may indicate an increased level of agitation, fear, or distress. Escape attempts and righting responses are also associated with distress [7,8]. Righting responses suggest an awareness of being physically impaired and can be distressing [18]. Since these distressful behaviors happened more frequently in the N_2_O treatment, it could be interpreted as CO_2_ being more humane. However, in the CO_2_ treatment, the process is a lot more rapid than the N_2_O treatment and it is more likely that the piglets did not have time to show these distressful behaviors before loss of posture. If the piglets lost posture at the same time in both treatments a better comparison could be made of these behaviors.

When comparing exposure to CO_2_ post-N_2_O against CO_2_ alone, all latencies took a shorter amount of time to occur in the post-N_2_O data. The sequence of behaviors occurred more rapidly, and piglets were not heavy breathing or open-mouth breathing for as long as piglets in the CO_2_ treatment. Gaping happened a lot sooner post-N_2_O compared to the CO_2_ treatment. Gaping are deep thoracic movements that once stopped, indicate respiratory arrest or last movement and are considered rudimentary brain stem reflexes [21]. Called gagging by Verhoeven et al., it is considered an indicator of a deep state of unconsciousness [14]. The time to the first piglet’s last movement and clinical death were also shorter post-N_2_O, indicating that the piglets in the N_2_O treatment spent less time in CO_2_ compared to the CO_2_ treatment.

Euthanizing piglets in groups allowed for more occurrences of the behaviors as there were more piglets to show the behaviors, which may be why there are differences in the groups compared to the individual data. Individuals had a significant difference in age and showed a trend for rooting bouts while groups showed a significant difference in squeals, escape attempts, CO_2_ total time, procedure total time, and a trend for righting responses. In the post-N_2_O data, individuals only showed significant differences for ataxia and loss of posture while the group data was significantly different for all latencies. There were still groups that did not show all the behaviors. Three groups did not squeal at all and eight groups had no escape attempts. This may be due to their age and older piglets may show greater or more pronounced differences. The difference between group and individual data may have been due to mixing stress in the groups, however, the piglets were very young, compromised, and only spent a short duration in the group; thus, they had little time to express dominance or agonistic behavior. No agonistic behaviors were observed in the behavior observations.

Although the N_2_O treatment did not reduce the amount of distressful behaviors overall, it did reduce the duration that groups of piglets had to be exposed to CO_2_. Swine exposed to CO_2_ concentrations at or greater than 20% [5] to 30% [6] find it highly aversive. Piglets in the N_2_O treatment were exposed to CO_2_ after they had lost posture. Loss of posture is an indicator of the onset of loss of consciousness [14]. If the piglets started to lose consciousness before experiencing heavy breathing and open-mouth breathing, they may not have been in as much distress. Further studies would need to be done to make any conclusions.

### 4.3. Shortcomings and Future Work

Most euthanasia studies have been conducted on healthy animals as they are readily available and provide a uniform experimental unit. This study used compromised piglets destined for euthanasia. The various causes that classified the piglets to be euthanized may have influenced the results. It was observed that piglets that were injured did not get around as easily and some of the piglets that were ill were less responsive.

Since older piglets take longer to be influenced by gases and show more distressful behaviors than neonates [8], a future experiment that uses older piglets would be beneficial. For producers, it would allow them to have the same procedure for neonates and weaned piglets when euthanizing. For the experimenter, it may show outcomes of greater significant differences and go on to further support that N_2_O is indeed more humane for piglets than CO_2_.

## 5. Conclusions

Euthanizing neonatal piglets by exposing them for six min to N_2_O resulted in piglets losing posture in the N_2_O before being exposed to CO_2_. In the N_2_O treatment, heavy breathing and open-mouth breathing only occurred after loss of posture and when being exposed to CO_2_, presumably after they had started to lose consciousness. Piglets euthanized with CO_2_ alone experienced the distressful effects of heavy breathing and open-mouth breathing before losing posture. However, groups of piglets in the N_2_O treatment had more time upright than the CO_2_ treatment which may have allowed them more time to display the increased number of squeals and escape attempts. Therefore, the results do not support the hypothesis that using N_2_O in a two-step process is more humane than CO_2_ alone.

## Figures and Tables

**Table 1:** Ethogram of piglet’s behavior during euthanasia for Experiments 1 and 2. Behavioral recordings started from the time the two straps of the box were latched. Interruptions shorter than 3 s are considered the same bout of behavior.

Category	Behavior	Description
Duration	Stand	Up on four legs.
	Lateral Lying	Lying down with side in contact with the floor.
	Ventral Lying	Lying down with sternum and belly in contact with the floor.
	Sit	One or two hind legs folded underneath the body and supporting weight on two front legs. “Sitting like a dog“.
	Kneel	One or two front legs folded underneath the body with hind legs straight.
	Locomotion	Any movement more than two steps; walk or run.
	Inactive	Immobile, not doing any particular behavior.
Latency	Ataxic	Lack of muscle coordination in basic movements, loss of balance on one or more feet.
	Loss of Posture ^2^	Piglet lies on the ground and does not get back up.
	Heavy Breathing ^2^	Forceful and quick repetition of flank movements, mouth closed.
	Open-Mouth Breathing ^2^	Mouth open to breath from.
	Gaping ^2^	Deep forceful, rhythmic movements of the chest with mouth open, a rudimentary brain stem reflex, deep state of unconsciousness.
	Last Movement ^2^	Piglet stops gaping and does not move at all, is clinically dead. End of experiment time.
	Paddle Bout ^1,2^	While lying laterally the piglet’s legs paddle/run.
Events	Rooting Bout ^1^	Snout in contact with the floor touching, sniffing, rubbing, or chewing.
	Escape Attempt ^1,2^	Rear on hind legs, jump (all limbs lose contact with the floor), or scratch with front legs against walls.
	Neck Stretch ^1^	Extend neck as much as possible, head up.
	Foot Slips ^1^	Piglet scrambles/loses balance while standing.
	Righting Response ^1,2^	Unsuccessful effort to right up onto four legs.
	Squeal ^1,2^	High-pitched vocalization; extended sound of high amplitude and frequency.
	Grunt ^1^	Low-pitched vocalization; sound of low to medium amplitude.
	Intermediate ^1^	Vocalization that starts out low-pitched and ends high-pitched. Neither a grunt or squeal but some combination.

^1^ Behaviors recorded as events due to their brief nature, rather than as states; ^2^ Behaviors recorded for Experiment 2.

**Table 2:** Data collected (mean ± SE) on individual pigs for the two treatments during Experiment 1.

Variable ^1^	CO_2_	N_2_O	*p*-Value
Day of Age	5.75 ± 0.37	4.00 ± 0.62	0.034 *
Body Temperature (°C)	37.85 ± 17.52	37.67 ± 17.60	0.562
Weight (kg)	0.96 ± 0.07	1.04 ± 0.08	0.412
CO_2_ Total Time (min)	14.88 ± 2.43	13.78 ± 2.58	0.410
Procedure Total Time (min)	14.57 ± 2.43	19.29 ± 0.75	0.457
Latency to no Palpebral Reflex (s)	313.38 ± 43.70	560.22 ± 26.63 ^2^	<0.001 *

^1^ s = seconds, min = minute, kg = kilogram; * *p* < 0.05 significant statistical difference. ^2^ This behavior was shown in piglets exposed to CO_2_ post-N_2_O.

**Table 3:** Duration and latency data (mean ± SE) on individual piglets during Experiment 1.

Category	Variable (s) ^1^	CO_2_	N_2_O	*p*-Value
Duration	Standing	99.81 ± 9.15	202.69 ± 51.45	0.946
	Lateral Lying	656.32 ± 149.02	766.13 ± 175.47 ^2^	0.630
	Ventral Lying	94.55 ± 40.09	164.06 ± 41.37 ^2^	0.491
	Sitting	26.47 ± 4.77	29.37 ± 18.51	0.123
	Kneeling	0.00 ± 0.00	3.38 ± 2.92	0.169
	Locomotion	19.99 ± 5.01	52.76 ± 18.84	0.336
	Inactivity	226.62 ± 105.24	231.60 ± 36.93 ^2^	0.149
Latency	Ataxia	81.43 ± 6.03	181.43 ± 36.25	0.021 *
	Heavy Breathing	61.61 ± 10.93	356.79 ± 31.29 ^2^	<0.001 *
	Open-Mouth Breathing	86.71 ± 9.82	439.87 ± 9.99 ^2^	0.001 *
	Gaping	143.57 ± 9.53	495.43 ± 13.95 ^2^	0.001 *
	Loss of Posture	125.16 ± 6.48	284.36 ± 43.29	0.004 *
	First Paddle Bout	616.88 ± 180.62	960.82 ± 197.94 ^2^	0.370

^1^ s = seconds; * *p* < 0.05 indicate significant statistical difference; ^2^ This behavior was shown in piglets exposed to CO_2_ post-N_2_O.

**Table 4:** Frequency of events collected (mean ± SE) on individual piglets during Experiment 1.

Variable (Frequency)	CO_2_	N_2_O	*p*-Value
Escape Attempts	1.38 ± 0.60	2.11 ± 1.51	0.499
Neck Stretches	4.38 ± 1.02	3.89 ± 2.30	0.143
Righting Responses	0.88 ± 0.40	2.33 ± 0.90 ^1^	0.249
Paddle Bouts	0.50 ± 0.27	0.67 ± 0.33 ^1^	0.784
Foot Slips	0.75 ± 0.49	4.33 ± 2.22	0.482
Rooting Bouts	1.25 ± 0.59	5.00 ± 1.91	0.095 ^†^
Squeals/Minute	0.12 ± 0.06	0.80 ± 0.46	0.587
Intermediates/Minute	1.02 ± 0.67	2.18 ± 1.11	0.381
Grunts/Minute	1.87 ± 0.40	4.89 ± 1.37	0.175

^†^ 0.05 < *p* < 0.10 trend, ^1^ This behavior was shown in piglets exposed to CO_2_ post-N_2_O.

**Table 5:** Event and miscellaneous data (mean ± SE) collected on groups of piglets during Experiment 2.

Variable ^1^	CO_2_	N_2_O	*p*-Value
Average Day of Age	2.28 ± 0.64	1.71 ± 0.45	0.480
Average Weight (kg)	0.67 ± 0.07	0.61 ± 0.03	0.825
Squeals/Minute	0.48 ± 0.25	4.90 ± 1.41	0.004 *
Escape Attempts/Piglet	0.17 ± 0.12	0.79 ± 0.32	0.021 *
Righting Responses/Piglet	0.36 ± 0.06	1.41 ± 0.39	0.084 ^†^
CO_2_ Total Time (min)	8.56 ± 0.18	6.56 ± 0.34	<0.001 *
Procedure Total Time (min)	11.56 ± 0.59	14.54 ± 0.50	0.001 *

^1^ min = minute, kg = kilogram; * *p* < 0.05 significant statistical difference; ^†^ 0.05 < *p* < 0.10 trend.

**Table 6:** Latency data (mean ± SE) on groups of piglets for Experiment 2.

Latency Variable (s) ^1^	CO_2_	N_2_O	*p*-Value
First Piglet Heavy Breathing	30.94 ± 2.15	364.46 ± 5.35 ^2^	<0.001 *
First Piglet Open-Mouth Breathing	39.94 ± 2.58	386.22 ± 4.44 ^2^	<0.001 *
First Piglet Gaping	88.13 ± 6.43	425.88 ± 4.63 ^2^	<0.001 *
All Piglets Gaping	115.73 ± 5.24	458.38 ± 5.76 ^2^	<0.001 *
First Piglet Loss of Posture	71.80 ± 5.13	191.50 ± 11.61	<0.001 *
Last Piglet Loss of Posture	105.65 ± 1.96	315.90 ± 11.61	<0.001 *
First Piglet’s Paddle Bout	278.26 ± 65.66	427.43 ± 17.09 ^2^	0.043 *
First Piglet’s Last Movement	353.05 ± 13.03	617.27 ± 13.36 ^2^	<0.001 *

^1^ s = seconds; * *p* < 0.05 significant statistical difference. ^2^ This behavior was shown in piglets exposed to CO_2_ post-N_2_O.

**Table 7:** Post-N_2_O vs. CO_2_ treatment latency data (mean ± SE) on groups of piglets for Experiment 1.

Latency Variable (s) ^1^	CO_2_	Post-N_2_O	*p*-Value
Ataxia	81.43 ± 6.03	−178.96 ± 36.25 ^2^	0.001 *
Heavy Breathing	61.61 ± 10.93	−3.21 ± 31.29 ^2^	0.102
Open-Mouth Breathing	86.71 ± 9.82	79.87 ± 9.99	0.401
Gaping	143.57 ± 9.53	135.43 ± 13.95	0.826
Loss of Posture	125.16 ± 6.48	−75.64 ± 43.29 ^2^	0.002 *
First Paddle Bout	616.88 ± 180.62	600.82 ± 197.94	0.564

^1^ s = seconds; * *p* < 0.05 significant statistical difference. ^2^ The negative value indicates that the behavior occurred while piglets were in N_2_O, prior to CO_2_.

**Table 8:** Post-N_2_O vs. CO_2_ treatment latency data (mean ± SE) on groups of piglets for Experiment 2.

Latency Variable (s) ^1^	CO_2_	Post-N_2_O	*p*-Value
1st Piglet Heavy Breathing	30.94 ± 2.15	4.46 ± 5.35	<0.001 *
1st Piglet Open-Mouth Breathing	39.94 ± 2.58	26.22 ± 4.44	0.017 *
1st Piglet Gaping	88.13 ± 6.43	65.88 ± 4.63	0.013 *
All Piglets Gaping	115.73 ± 5.24	98.38 ± 5.76	0.041 *
1st Piglet‘s Loss of Posture	71.80 ± 5.13	−168.50 ± 11.61 ^2^	<0.001 *
Last Piglet‘s Loss of Posture	105.65 ± 1.96	−44.10 ± 11.61 ^2^	<0.001 *
1st Piglet’s Paddle Bout	278.26 ± 65.66	67.43 ± 17.09	0.001 *
1st Piglet’s Last Movement	353.05 ± 13.03	257.27 ± 13.36	<0.001 *

^1^ s = seconds; * *p* < 0.05 significant statistical difference. ^2^ The negative value indicates that the behavior occurred while piglets were in N_2_O, prior to CO_2_.
